# Personality interferences in the pathology of breast cancer: a cross-sectional single-center study

**DOI:** 10.25122/jml-2024-0302

**Published:** 2024-06

**Authors:** Andra Oltean, Andrei Manea, Aurel Nireştean, Raluca Niculescu, Mircea Gîrbovan, Elena Gabriela Strete

**Affiliations:** 1 Department of Psychiatry, Clinical County Hospital, Targu Mureş, Romania; 2 Department of Radiology, Emergency Clinical County Hospital, Targu Mures, Romania; 3 Department of Psychiatry, George Emil Palade University of Medicine, Pharmacy, Sciences and Technology, Targu Mures, Romania; 4 Department of Pathophysiology, George Emil Palade University of Medicine, Pharmacy, Science, and Technology, Targu Mures, Romania; 5 Department of Urology, Clinical County Hospital, Targu Mureş, Romania

**Keywords:** personality dimensions, breast cancer, quality of life, DECAS Personality Inventor, DECAS, Big-Five Personality Inventory, D, Openness, E, Extroversion, C, Conscientiousness, A, Agreeableness, S, Emotional Stability, ICD, International Classification of Diseases, DSM, Diagnostic and Statistical Manual of Mental Disorders, TP, Personality Disorder, TPS, Schizoid Personality Disorder, Schizotypal TP, Schizotypal Personality Disorder, TPP, Paranoid Personality Disorder, TPN, Narcissistic Personality Disorder, TPA, Antisocial Personality Disorder, TPB, Borderline Personality Disorder, TPHy, Hysterical Personality Disorder, TPE, Anxious Avoidant Personality Disorder, TPD, Dependent Personality Disorder, TPOC, Obsessive-Compulsive Personality Disorder

## Abstract

Individual personality refers to the Ego and the interpersonal sector. The Ego corresponds to consciousness and self-esteem, including the capacities for emotional self-regulation, self-control, self-evaluation, and self-direction in relation to personal goals. When neoplastic and psychiatric diseases coexist, a patient's quality of life is significantly impacted. While there are somatic differences in disease progression, how the illness is perceived and mainly experienced depends on personality traits. In this study, we administered the DECAS Personality Inventory (a Romanian-validated instrument based on the Five-Factor model of personality) to a group of 121 patients diagnosed with breast cancer to explore the relationships among their personality traits. Descriptive statistics revealed that the mean T scores for openness, extroversion, and emotional stability were low, while the scores for conscientiousness and agreeableness were at an average level. Our findings suggest that, in the studied group, low levels of emotional stability, extroversion, and openness were unfavorable personality dimensions that should be a primary focus of therapeutic strategies, as they significantly affect the quality of life in patients with breast cancer.

## INTRODUCTION

Disease can be defined as the totality of statistical deviations from normality—assessed from somatic, psychological, and social perspectives—that either alters an individual’s ability to function in all areas of life (personal, professional, or social) or pose a significant risk of such impairment due to their rapid progression [1].

In contemporary society, psychiatric disorders are notable not only as distinct conditions but also for their frequent comorbidities, particularly with neoplastic diseases [2]. Breast cancer, similar to other debilitating diseases, has a profound impact on the psyche and individual personality traits. The chronic nature of the disease, along with the emergence of various complications, often leads to disruptions in self-image and self-esteem. These challenges are closely linked to difficulties in fulfilling life roles and maintaining interpersonal relationships. Moreover, psychological factors play a significant and proportional role in influencing the progression and prognosis of related somatic conditions [3,4].

Human personality is a complex of values, goals, and experiences reflected in behavior across various life roles. Personality traits are flexible and adaptable, allowing individuals to function and integrate into social life [5]. However, maladaptive personality traits result from negative life experiences and can influence the onset and evolution of a variety of psychopathological disorders [6].

The progression of a disease can vary from a physical standpoint, but how it is perceived and experienced is largely influenced by individual personality traits [7]. When both neoplastic and psychiatric conditions are present, the patient’s quality of life tends to deteriorate significantly, necessitating the involvement of skilled professionals [8].

According to the International Classification of Diseases (ICD-10), personality disorders are described as patterns of maladaptive behavior that become evident in adolescence and may become less pronounced later in life. The structural abnormality of personality lies in the imbalance between its different components, their qualitative alteration, and the deterioration of their expression in individual behavior [9].

The Diagnostic and Statistical Manual of Mental Disorders (DSM-5) defines pathological personalities as anomalies of the Ego, encompassing consciousness, self-esteem, self-evaluation, self-control, emotion regulation, and self-direction, all of which affect the accuracy of personal goal formulation [8,9].

Personality can be assessed both dimensionally and categorically. Personality disorders are a distinct nosological category characterized by pathological features that create an early and persistent adaptive deficit throughout life, often associated with feelings of unfulfillment and reduced capacity for personal satisfaction. This categorical perspective aligns with the definition of personality disorders [10,11]. The diagnosis of a personality disorder is based on specific criteria outlined in the DSM-IV-TR, which categorizes ten pathological personalities into three clusters: TPS (schizoid), schizotypal TP, TPP (paranoid), TPN (narcissistic), TPA (antisocial), TPB (borderline), TPHy (hysterical), TPE (anxious-avoidant), TPD (dependent), and TPOC (obsessive-compulsive) [8,9,12].

The dimensional perspective, developed in response to the limitations of the categorical approach, allows for the assessment and understanding of personality on a continuum between normality and pathological traits. This approach highlights both adaptive and maladaptive traits in individuals [13]. The most well-known dimensional approach is the Five-Factor Model, described by Costa in 1985, which identifies five major dimensions: openness, extroversion, conscientiousness, agreeableness, and emotional stability [14].

Both disharmonious and normal personality traits are unconscious or partially conscious and stable over time. Lenzenweger [15] and Cloninger *et al*. [16] describe how genes are responsible for persistent personality traits related to temperament, while the socio-cultural environment, which is less stable, is shaped by education, socialization, and involvement in life roles.

Viewing personality dimensions as a complex of cognitive, affective, relational, motivational, and spiritual attributes allows for a more nuanced understanding of the dynamic structure of personality [17]. Since personality traits and dimensions play a fundamental role in shaping the course of any disease, it is essential to assess them in conditions like breast cancer, which profoundly affects both the clinical outlook and the overall experience of the patient. The primary objective is to identify any direct relationships between the clinical and developmental aspects of breast cancer and individual personality traits, which could help pinpoint psychological vulnerabilities and resilience factors. This knowledge can enhance the complexity and effectiveness of therapeutic strategies [18].

Our study aimed to explore potential correlations between the Big Five personality traits and the psychological profiles of women diagnosed with breast cancer.

## MATERIAL AND METHODS

This cross-sectional, prospective, single-center study was conducted between August 1, 2023, and May 1, 2024. The study included 150 patients diagnosed with breast cancer at Mureş County Clinical Hospital, all of whom completed the DECAS personality traits questionnaire.

### Participants and procedure

Out of the initial 150 patients interviewed using the DECAS questionnaire, only 121 met all the eligibility criteria based on the inclusion and exclusion criteria.

**Inclusion criteria**: Breast cancer diagnosis, female patients aged between 18 and 75 years.

**Exclusion criteria**: Presence of comorbidities with a significant impact on mental health, diagnosis of personality disorders, or chronic psychiatric conditions.

### Measures

The DECAS personality traits assessment tool, based on the Big Five Factors Model developed by Prof. Dr. Florin Alin Sava, consists of 97 true/false questions. It assesses five personality dimensions: openness, extraversion, conscientiousness, agreeableness, and emotional stability, each with six facets described by Costa and McCrae in 1985 [19].

Regarding the psychometric properties of the inventory, internal consistency was calculated from a sample of 1,524 individuals. The Cronbach's alpha coefficient for the Romanian population ranged from 0.70 for conscientiousness to 0.75 for emotional stability. Specifically, the internal consistency coefficient was 0.69 for openness and 0.71 for agreeableness [19].

According to the DECAS manual, the dimensions can be characterized as follows:

### Openness (D)

**High (D**↑**)**: Unconventional, eccentric, detached from reality, unpragmatic, hypersensitive, lacking coherent beliefs and values.

**Low (D**↓**)**: Rigid, apathetic, unimaginative, struggles to adapt to new situations, lacks empathy.

### Extraversion (E)

**High (E**↑**)**: Attention-seeking, superficial, risk-averse, difficulty relaxing, easily fatigued, hyperreactive to minor events.

**Low (E**↓**)**: Anhedonic, emotionally detached, passive, sedentary, easily bored, struggles with emotional regulation.

### Conscientiousness (C)

**High (C**↑**)**: Rigid, meticulous, perfectionistic, ambivalent, lacks spontaneity, strictly adheres to norms and rules, struggles to adapt to new situations, highly responsible.

**Low (C**↓**)**: Careless, disorganized, inefficient, irresponsible, lacks purpose, immoral, duplicitous.

### Agreeableness (A)

**High (A**↑**)**: Generous, tolerant, docile, empathetic, overly vulnerable due to ignoring self-interest.

**Low (A**↓**)**: Cynical, arrogant, greedy, suspicious, physically and verbally aggressive.

### Emotional Stability (S)

**High (S**↑**)**: Emotionally flat, rigid, indifferent, overestimates abilities, easily bored.

**Low (S**↓**)**: Anxious, sad, low self-control, pessimistic, emotionally unstable, helpless, prone to substance abuse and suicidal ideation.

The DECAS test results are interpreted using the T score as a standard measure. The dimensions can be quantitatively classified as very low (T scores between 20.00 and 34.99), low (T scores between 35.00 and 44.99), average (T scores between 45.00 and 55.00), high (T scores between 56.01 and 65.99), and very high (T scores between 66.00 and 80.00).

### Statistical analysis

Statistical analysis was performed using IBM SPSS 26 software, and data manipulation was conducted using Microsoft Excel 365. The significance level for the *P* value was set at 0.05, with a confidence interval (CI) of 95%. The statistical analysis included descriptive statistics (mean, standard deviation) and inferential statistics. The Shapiro-Wilk test was used to assess the data distribution. The Spearman test was employed to measure the strength and direction of the association between numerical variables.

## RESULTS

Out of the initial 150 subjects, 121 women met the eligibility criteria for the study, with a mean age of 61.46 ± 5.81 years. Most participants resided in an urban area (*n* = 90, 74.4%), and 31 (25.6%) were from rural areas. Regarding their professions, the group was divided into six categories: administration and support (13 subjects, 8.84%), healthcare (39 subjects, 26.53%), commerce and services (6 subjects, 4.08%), agriculture and food (6 subjects, 4.08%), education (9 subjects, 6.12%), and retirees (48 subjects, 32.65%). The demographic characteristics are summarized in Table 1.

**Table 1:** Demographic characteristics of participants

Sample characteristics	*n* = 121
Age range M (SD)	61.46 (5.81)
Place of residence, *n* (%)	
Urban Rural	90 (74.4%) 31 (25,6%)
Workplace, *n* (%)	
Administration and support	13 (8.84%)
Healthcare	39 (26.53%)
Commerce and services	6 (4.08%)
Agriculture and food	6 (4.08%)
Education	9 (6.12%)
Retiree	48 (32,65%)

Descriptive statistics revealed that the mean T scores for openness (38.88 ± 5.05), extraversion (40.17 ± 7.82), and emotional stability (41.26 ± 3.55) were classified as low (T scores between 35.00 and 44.99) (Table 2). Moreover, the scores for conscientiousness (46.64 ± 6.85) and agreeableness (46.74 ± 5.12) were at the lower end of the average range (T scores between 45.00 and 55.00), according to the DECAS manual.

**Table 2:** Descriptive statistics of DECAS score

Variable	M	SD	S.E.	95% CI for Mean	
				Lower Bound	Upper Bound
Openness	38.88	5.05	0.45	37.972	39.792
Extraversion	40.17	7.82	0.71	38.767	41.585
Conscientiousness	46.64	6.85	0.62	45.407	47.876
Agreeableness	46.74	5.12	0.46	45.824	47.670
Emotional Stability	41.26	3.55	0.32	40.622	41.902

M, mean; SD, standard deviation, S.E, standard error, CI, Confidence Interval

We assessed the correlations between each DECAS score using the Spearman test, as the scores did not pass the normality test, indicating a non-Gaussian distribution. We found significant correlations between openness and extraversion (*r* = 0,242, *P* = 0.007; weak positive correlation), openness and emotional stability (*r* = 0.550, *P* < 0.0001; moderate positive correlation). Extraversion was negatively correlated to conscientiousness (*r* = -0.469, *P* < 0.0001; moderate negative correlation) and positively correlated to emotional stability (*r* = 0.446, *P* < 0.001; moderate positive correlation). Conscientiousness was negatively correlated with agreeableness (*r* = -0.315, *P* < 0.001; weak negative correlation). All the correlations presented were statistically significant, as the *P* value was less than 0.05. The correlations between each of the DECAS scores are detailed in Table 3.

**Table 3:** Spearman correlation coefficients between DECAS personality dimensions

	Openness			Extraversion			Conscientiousness			Agreeableness			Emotional Stability		
	*r*	*P*	95 % CI	*r*	*P*	95 % CI	*r*	*P*	95 % CI	*r*	*P*	95 % CI	*r*	*P*	95 % CI
Openness				0.242**	0.007	0.043 to 0.423	0.049	0.593	-0.162 to 0.226	0.083	0.364	-0.123 to 0.283	0.550**	<0.001	0.384 to 0.687
Extraversion	0.242**	0.007	0.043 to 0.423				-0.469**	<0.001	-0.594 to – 0.319	-0.154	0.093	-0.326 to 0.028	0.446**	<0.001	0.266 to 0.614
Conscientiousness	0.049	0.593	-0.162 to 0.226	-0.469**	<0.001	-0.594 to – 0.319				-0.315**	<0.001	-0.477 to -0.157	-0.065	0.480	-0.24 to 0.119
Agreeableness	0.083	0.364	-0.123 to 0.283	-0.154	0.093	-0.326 to 0.028	-0.315**	<0.001	-0.477 to – 0.157				0.209*	0.022	0.007 to 0.39
Emotional Stability	0.550**	<0.001	0.384 to 0.687	0.446**	<0.001	0.266 to 0.614	-0.065	0.480	-0.24 to 0.119	0.209*	0.022	0.007 to 0.39			

r, correlation coefficient; *Correlation is significant at the 0.05 level (2-tailed), **Correlation is significant at the 0.01 level (2-tailed).

## DISCUSSION

The low levels of the openness dimension observed in the mean score suggest maladaptive traits such as rigidity, lack of imagination, and deficits in volition and motivation. These characteristics are disadvantageous for patients coping with illness, as they can exacerbate subjective distress and hinder therapeutic compliance [20]. Most patients in our study presented low values of extraversion, which, corresponding to passivity and affective detachment, as well as low needs for expression and socialization, make them vulnerable to suffering and favor anxious and ambivalent ideo-affective experiences. In the same context are the diminished values of emotional stability that maintain an affective tone dominated by anxiety, negative anticipations, and a feeling of incompleteness. Furthermore, due to the low control of the impulses, a suicidal potential can emerge, these characteristics simultaneously affecting the subjective well-being and the individual well-being. There is evidence of a statistically significant association between low extraversion levels and shorter survival in breast cancer patients [21,22]. Conversely, higher extraversion scores have been linked to increased use of healthcare services following a breast cancer diagnosis [23,24]. An Eysenck Personality Inventory (EPI) study showed that patients with breast cancer and higher extraversion scores tended to have a lower risk of death [3].

We found a weak negative correlation between agreeableness and conscientiousness in the studied group. These traits may serve as protective factors that enhance resilience and stability in therapeutic relationships. These two dimensions can also have an adaptive role through the progressive development of adaptation mechanisms based on a territory laden with suffering and deep existential significance. Patients with high agreeableness are more likely to have a better quality of life [25]. They create positive interactions with medical services and adhere to their treatment [26].

The positive correlation between openness, extraversion, and emotional stability in the study group suggests that these three personality dimensions are interconnected in patients with neoplastic diseases such as breast cancer. Bahat's study supports this finding, indicating that high openness, extraversion, and conscientiousness predict better patient compliance and greater participation in breast self-examination [27]. A study that evaluated the level of information about breast self-examination among medical students showed that more than 90% think that this procedure is helpful for early cancer detection. Most participants (71%) knew breast self-examination and 60% knew how it must be performed, but only 16% of all performed it regularly [28].

Conscientiousness was found to correlate negatively with extraversion, indicating that patients with higher extraversion levels tend to be less conscientious than those with lower extraversion levels. Similarly, conscientiousness negatively correlated with agreeableness, suggesting that more conscientious patients may display lower levels of agreeableness toward others.

### Limitations

Our study had some limitations that warrant consideration in future research. The primary limitation was self-administered questionnaires, which could have led to inaccuracies, as only the DECAS Personality Inventory includes an internal validation scale capable of detecting distorted responses.

## CONCLUSION

Low levels of openness, extraversion, and emotional stability in oncology patients, particularly those diagnosed with breast cancer, are considered predisposing factors for the development of psychopathological episodes. These personality dimensions in our study group emphasize the vulnerability of the patients to psychological illness, especially given the significant impact of a breast cancer diagnosis. In conclusion, within the studied group, low levels of openness (D), extraversion (E), and emotional stability (S) are unfavorable dimensional markers that should be prioritized in therapeutic strategies. The ultimate goal of psychosocial interventions, regardless of the type, orientation, or stage at which they are applied, remains consistent: to help patients acquire the skills and resources necessary to cope with their illness while enhancing their quality of life.

## Data Availability

Further data is available from the corresponding author upon reasonable request.
